# LGI1 encephalitis: potentially complement-activating anti-LGI1-IgG subclasses 1/2/3 are associated with the development of hippocampal sclerosis

**DOI:** 10.1007/s00415-024-12594-9

**Published:** 2024-08-06

**Authors:** Christian G. Bien, Anna Rada, Markus Mertens, Corinna I. Bien, Jan Bauer, Anne Hagemann, Friedrich G. Woermann

**Affiliations:** 1 Department of Epileptology, Krankenhaus Mara, Bethel Epilepsy Center, Medical School OWL, Bielefeld University, Maraweg 21, 33617 Bielefeld, Germany; 2 Laboratory Krone, Bad Salzuflen, Germany; 3 Society for Epilepsy Research, Bielefeld, Germany; 4 Department of Neuroimmunology, Center for Brain Research, Medical University of Vienna, Vienna, Austria

**Keywords:** LGI1 antibodies, Immunoglobulin G subclasses, Hippocampal sclerosis, Risk factors

## Abstract

Two-thirds of published patients with anti-leucine rich, glioma inactivated 1 (LGI1) encephalitis develop hippocampal sclerosis (HS). It is likely that this contributes to residual cognitive long-term deficits and the risk of epilepsy. Almost all patients harbor anti-LGI1-immunoglobulin G-(IgG-) subclass 4, which is considered a “benign”, non-destructive subclass. In contrast, neuropathological case studies have suggested that the classical complement cascade may contribute to mediotemporal cell death in patients with LGI1 antibodies. IgG subclasses 1, 2, or 3 are required to initiate this cascade. We hypothesized that patients with these anti-LGI1-IgG1/2/3 in addition to IgG4 have a higher risk of developing HS than patients with anti-LGI1-IgG4 alone. We retrospectively assessed all anti-LGI1 encephalitis patients from this center with anti-LGI1-IgG-subclass information and follow-up MRI available. Nine out of 20 patients had developed HS (45%). Volumetric FreeSurfer analysis confirmed the visual HS diagnoses. HS and a lower hippocampal volume were associated with anti-LGI1-IgG1/2/3. All six patients with this IgG subclass status developed HS. There was no association with older or younger age at onset, female sex, longer latency from disease onset to start of immunotherapy, less intense immunotherapy, higher serum titers of LGI1 antibodies, LGI1 antibodies in CSF or higher LGI1-specific antibody indices. There was no association between anti-LGI1-IgG1/2/3 status and neuropsychological performance, epilepsy, or general neurological performance. This confirms our hypothesis that anti-LGI1-IgG1/2/3 in serum puts patients at risk of developing HS. If these findings can be confirmed and clinically corroborated, patients with anti-LGI1-IgG1/2/3 might become candidates for anti-complement-directed immunological treatments.

**Supplementary Information:**

The online version contains supplementary material available at 10.1007/s00415-024-12594-9.

## Introduction

Anti-leucine-rich, glioma-inactivated protein 1 (LGI1) encephalitis is one of the more common forms of autoimmune encephalitis. It mostly manifests as faciobrachial dystonic seizures (FBDS) [45] or limbic encephalitis [1, 32, 47]. Enduring structural hippocampal damage (in the literature termed “hippocampal atrophy”, “hippocampal sclerosis” [HS] or “hippocampal lesion”) is a complication of the condition. According to the existing series, 47–96% of patients develop hippocampal structural damage after median follow-ups of 12–40 months (pooled data: 127/199, 64%) [7, 12, 17, 27, 32, 35, 42, 44]. Neuropathological case studies have documented hippocampal nerve cell loss and gliosis in anti-LGI1 limbic encephalitis [4, 11, 25, 40], i.e., HS [6]. It is plausible that this structural hippocampal damage underlies the residual memory deficits after the active phase of anti-LGI1 encephalitis [12] and possibly the rare development of epilepsy [17].

The following pathogenic mechanism of LGI1 antibodies has been established: in synapses containing presynaptic Kv1.1 potassium channels and postsynaptic α-amino-3-hydroxy-5-methyl-4-isoxazolepropionic acid receptors (AMPAR), LGI1 antibodies disrupt the interaction between the secreted protein LGI1 and postsynaptic ADAM22 as well as LGI1 and presynaptic ADAM23. This disruption leads to a reduction of Kv1.1 potassium channels and AMPAR densities and thereby causes (i) neural hyperexcitability manifesting as epileptic seizures and (ii) decreased plasticity resulting in memory deficits [34, 35, 38]. Others have observed the internalization of the LGI1-ADAM22/23 complex [38]. Seizures and memory deficits usually improve with immunotherapy and sometimes in parallel to falling antibody titers [37]. It is, therefore, plausible that these pathomechanisms underlie the remediable aspects of anti-LGI1 encephalitis.

For irreversible structural mediotemporal damage and residual cognitive deficits, another mechanism has been described: the classical complement cascade leading to activation of the terminal membrane attack complex, which permits water influx into the target cell and its subsequent death. C9neo, indicative of functional activation of the complement cascade, as well as signs of cell death have been observed in the mediotemporal neurons of two patients with anti-LGI1 limbic encephalitis [4, 25]. Confirmatory evidence for hippocampal and amygdalar C9neo deposition comes from spontaneous limbic encephalitides with LGI1 antibodies in five cats [46]. Complement activation by LGI1 antibodies has also been demonstrated *in vitro* [20]. This mechanism, however, seems at odds with the observation that almost all patients with anti-LGI1 encephalitis harbor anti-LGI1-immunoglobulin G-(IgG-)4 antibodies [1, 3, 14, 31], so that anti-LGI1 encephalitis has been subsumed under the group of IgG4-mediated neurological autoimmune disorders [19]. IgG4 antibodies are functionally monovalent and bispecific. They are well suited to inhibiting protein-protein-interactions [19], but they do not or only minimally bind C1 as the starter of the classical complement cascade [5, 10, 41]. Complete IgG antibodies of the subclasses 1, 2, or 3 (IgG1/2/3), however, can bind C1 in the order of strength IgG3>IgG1>>IgG2 [2, 21]. In fact, serum anti-LGI1-IgG1/2/3 were found in a relevant proportion of patients by four studies [1, 3, 14, 31], see Table 1.

**Table 1:** Frequency of anti-LGI1-IgG subtypes in the literature and in the present study

	IgG1 *n*(%)	IgG2 *n*(%)	IgG3 *n*(%)	IgG4 *n*(%)
Ariño 2016 [1], *n* = 57	10 (18)	24 (42)	0	57 (100)
Gadoth (2018) [14], *n* = 23	6 (26)	0	0	23 (100)
Bien 2020 [3], *n* = 75	22 (29)	18 (24)	1 (1)	69 (92)
Muñiz-Castrillo 2021 [31], *n* = 89	77 (87)	26 (30)	14 (16)	72 (81)
Present study, *n* = 20	5 (25)	3 (15)	2 (10)	20 (100)

Sums are > 100% because patients may harbor more than one subclass

*IgG* immunoglobulin G, *LGI1* leucine-rich, glioma inactivated protein 1

We hypothesized that patients harboring anti-LGI1-IgG1/2/3 might be at an increased risk of developing HS and having lower hippocampal volumes. Alternative hypotheses were that predictors of a (differently defined) “poor outcome” might also be associated with the development of HS and lower hippocampal volumes. These factors are: older [24, 31] or younger age at onset [44], female sex [31, 44], longer duration between disease onset and the start of immunotherapy [12, 17, 45], less intense immunotherapy [43], higher titers of serum LGI1 antibodies [37], LGI1 antibodies in the CSF [24, 31], and higher specific antibody indices indicating the intrathecal synthesis of LGI1 antibodies [14]. In addition, we tested the hypothesis that a mediotemporal lesion on the earliest available MRI predicts the development of HS.

## Patients and methods

We retrospectively identified all patients with anti-LGI1 encephalitis who were examined and treated between 2011 and 2023 in The Mara and included those with this minimum set of information: anti-LGI1-IgG subclasses in serum at first visit and a follow-up MRI.

### Clinical data

The dates of birth, disease onset, making the diagnosis of anti-LGI1 encephalitis and start and types of immunotherapies, as well as antibody titers, were obtained from hospital records. For bivariate associations, the titers were expressed as log2 values to reflect the dilution series, which were multiples of 1:2.

Patients were invited for a prospective outpatient follow-up visit with co-author AR, including brain MRI and neuropsychological investigation. For patients who did not attend, the most recent follow-up data from clinical visits were used. As neuropsychological outcome measures, we noted the results of each patient’s most recent Verbal Learning and Memory Test (VLMT, a derivate of the Rey Auditory Verbal Learning and Memory Test [RAVLT]) [18] with the domains learning (d5, d1–5) and free recall (d7 and d5–d7) in the form of raw values. This type of verbal memory test has proven useful in the cognitive assessment of patients with limbic encephalitis and LGI1 antibodies [12, 50]. We also checked whether epilepsy according to our recent definition [36] was present at most recent follow-up. Co-author AR determined the modified Rankin Score (mRS) [15] and the Clinical Assessment Scale in Autoimmune Encephalitis (CASE) [26] at follow-up before knowing the anti-LGI1-IgG1/2/3 subclass status.

### Antibody diagnostics, IgG subclass determination

LGI1 antibody titers in serum and CSF were those of the original clinically motivated investigation of the earliest available samples. Antibodies were detected using commercially available biochips (Euroimmun, Lübeck, Germany). These are assemblies of multiple cell-based assays (CBA) consisting of human embryonic kidney (HEK-293) cells, including cells transfected with a plasmid encoding for LGI1. These were fixed with paraformaldehyde. The indirect immunofluorescence protocol followed the manufacturer’s recommendations (Euroimmun, FA 112d-1005-6, IgG) with modifications: buffer: phosphate-buffered saline (PBS); patient serum diluted to 1:20, CSF undiluted; secondary antibody I (for sensitivity): biotinylated goat-anti-human IgG heavy and light chain (H+L, Jackson ImmunoResearch 109-065-088), 1:100, incubation time 30 min at room temperature (RT), subsequently visualized by incubation with streptavidin coupled with Alexa 594 (Jackson ImmunoResearch 016-580-084), 1:400, 30 min, RT; secondary antibody II (for specificity, simultaneous application with anti-IgG-HL): goat-anti-human antibody against the Fcγ fragment of IgG, conjugated with Alexa Fluor 488 (Jackson Immunoresearch 109-545-098) 1:200, 30 min, RT; nuclear counterstaining with Hoechst 33342, 1:10000; embedding with 1,4-Diazabicyclo[2.2.2]octan. Experienced technicians endpoint-titrated LGI1 antibodies with the anti-Fc secondary antibody in multiples of 1:2. The results were checked by an experienced neurologist (CIB or CGB) and, if necessary, corrected. As different dilution steps were used over the study period, the results were not multiples of each other throughout. We repeated the titration of five LGI1 antibody-positive sera, determined originally ten years ago and stored at – 20 ℃, and obtained results with ≤1 dilution stage difference. This confirmed the stability of the antibodies and consistency of the investigators’ sensitivity.

As part of the diagnostic process, samples were also tested at 1:40 (serum) and 1:2 (CSF) by default on a tissue-based assay in form of unfixed sagittal mouse brain slices containing hippocampus, brain stem, and cerebellum (Euroimmun, Lübeck, Germany) to detect a confirmatory LGI1 neuropil staining [33]. The protocol was identical to that for the CBA.

Anti-LGI1-IgG subclasses in serum were determined in 2023 by CBA with the following secondary antibodies used in a previous study [3]: biotinylated mouse anti-human-IgG1 (Biozol NMB-MAHU-IGG1-BIO), 1:20, 1 h, RT; biotinylated mouse anti-human-IgG2 (Biozol NMB-MAHU-IGG2-BIO), 1:200, 1 h, RT; biotinylated mouse anti-human-IgG3 (Sigma-Aldrich B3523), 1:200, 1 h, RT; biotinylated mouse anti-human-IgG4 (Sigma-Aldrich B3648), 1:200, 1 h, RT. All were subsequently visualized with streptavidin-Alexa 594, 1:200, 30 min, RT. In four cases, not enough serum was left for renewed subclass determination. In those cases, we used the subclass results of a previous study [3], determined in 2017. Even if enough CSF was available, there were hardly any positive IgG subclass stainings in our hands, so we did not include anti-LGI1-IgG subclasses in CSF in this study.

Specific LGI1-antibody indices were calculated according to the following formula; in theory, values >1 would suggest intrathecal synthesis, but for reasons of specificity, a cutoff >4 with the use of titers has been suggested [39]:

$$\frac{\left(\frac{LGI1\, abs \,in\, CSF}{LGI1\, abs\, in\, serum}\right)}{\left(\frac{IgG\, total\, in \,CSF}{IgG\, total\, in\, serum}\right)}$$

### MRI assessment

The diagnoses “HS” or “no HS” were taken from FGW´s original clinical readings of the individual patient’s most recent MRIs performed at our center (3 Tesla Siemens Magnetom Verio or Vida). The visual diagnosis of HS relied on a reduced hippocampal volume and abnormally high signal on FLAIR/T2 images [48]. FGW re-checked his diagnoses for the purposes of the study and confirmed all of them. Using 3D T1 weighted magnetization-prepared rapid gradient echo (MPRAGE, TR 1900 msec, TE 3 msec, TI 900 msec; voxel dimensions 0.8 x 0.8 x 0.8 mm^3^) scans of the most recent available MRI studies, hippocampal volumes were automatically segmented (FreeSurfer, version 7.3.2, standard analysis [13]). We added the volumes of left and right hippocampi of the individual patients and divided the result by two to obtain one mean hippocampal volume per patient.

### Statistics

The Fisher’s exact test and Mann–Whitney *U* test were used for group comparisons. Bivariate associations were calculated as Pearson correlation (*r*), point–biserial correlation (*r*_pbis_) or Phi coefficient depending on the constellation of variables. Statistical analyses were performed using IBM SPSS Statistics (version 29) and R software (version 4.3.0, package “epiR”). The significance level was set to *α* = 0.05.

### Ethics

The Ethics committee of the University of Münster approved the study (2020-244-f-S) and patients gave informed consent for the use of their data. In the case of those who could not be reached for consent, the Ethics committee waived it in accordance with the Gesundheitsdatenschutzgesetz Nordrhein-Westfalen (North-Rhine-Westphalian law of healthcare data protection) because it was a retrospective data assessment of personally studied patients.

## Results

We identified 26 patients with anti-LGI1 encephalitis. Two patients were excluded because of missing serum for subclass testing, one because the patient did not return for follow-up (so the MRI outcome was not known) and another three patients with serum LGI1 antibody titers <1:100 because their sera did not give any signal upon anti-LGI1-IgG subclass 1–4 testing. This left 20 patients (nine females) for the study with LGI1 antibody serum titers between 1:160 and 1:8000. Among them, 16 patients had confirmatory serum or CSF neuropil staining on the tissue-based assay or were CBA-positive with CSF, as required by authorities in the field [9]. Four patients [nos. 8 (serum titer 1:160), 14 (1:320), 19 (1:160) and 20 (1:160)] did not fulfill these criteria, but the LGI1 antibody positivity was corroborated by the IgG4 positivity, which is unlikely to occur by chance since the IgG4 subclass is estimated as making up for only 5% of the total amount of IgG [23]; more importantly, no better diagnosis than anti-LGI1 encephalitis could be made after a full diagnostic work-up (nos. 14 and 20: FBDS; nos. 8 and 19: limbic encephalitis). Sixteen patients had a prospective follow-up with AR. The individual patient data are summarized in Table 2 and Supplementary Table 1. Immunotherapy regimens are described in Supplementary Information 1.

**Table 2:** Patients’ core data

Pat. no	IgG1	IgG2	IgG3	IgG4	LGI1 ab serum titer	LGI1 ab CSF titer	Specific LGI1 ab index	MTL lesion on earliest MRI	HS at follow-up	Hipp. Volume at follow-up (mm^3^)	mRS at follow-up	CASE at follow-up
1	+	-	-	+	8000	16	0.8	R	R	2808	0	0
2	+	+	+	+	2560	2	0.6	L	L	3527	1	3
3	+	-	-	+	1280	8	2.2	Bil	L	3848	0	0
4	+	-	-	+	640	4	4.0	R	R	3608	2	4
5	-	+	-	+	375	8	3.3	L	L	3438	0	0
6	+	+	+	+	250	1	0.9	None	L	3836	0	0
7	-	-	-	+	160	4	1.6	R	Bil	3725	1	1
8	-	-	-	+	160	n.d	n.a	L	Bil	2917	0	0
9	-	-	-	+	160	4	4.0	None	R	3530	2	1
10	-	-	-	+	1280	8	1.5	None	None	3861	1	2
11	-	-	-	+	640	16	4.3	R	None	4774	0	0
12	-	-	-	+	640	2	1.0	None	None	3761	0	0
13	-	-	-	+	640	n.d	n.a	R	None	4291	2	2
14	-	-	-	+	320	0	0.0	None	None	4375	0	0
15	-	-	-	+	320	8	8.3	R	None	5106	2	5
16	-	-	-	+	320	2	1.7	R	None	4228	0	0
17	-	-	-	+	320	16	5.6	None	None	4139	2	4
18	-	-	-	+	320	1	1.3	R	None	3923	2	4
19	-	-	-	+	160	0	0.0	R	None	3884	0	0
20	-	-	-	+	160	0	0.0	None	None	4056	0	0
*Med*	*5* +	*3* +	*2* +	*20* +	*320*	*4*	*1.5*	*9 R* *3 L* *1 Bil*	*3 R* *4 L* *2 Bil*	*3854*	*0*	*0*
*Min*					*160*	*0*	*0.0*			*2808*	*0*	*0*
*Max*					*8000*	*16*	*8.3*			*5106*	*2*	*5*
*Mean*					*935*	*6*	*2.3*			*3882*	*1*	*1*
*SD*					*1759*	*6*	*2.2*			*542*	*1*	*2*

Sorted by (i) HS yes/no, (ii) LGI1 ab titer in serum

*ab* antibody, *Bil* bilateral, *CASE* Clinical Assessment Scale in Autoimmune Encephalitis, *Hipp*. Hippocampal, *HS* hippocampal sclerosis, *IgG* immunoglobulin G, *L* left, *LGI1* leucine-rich, glioma inactivated protein 1, *Max* maximum, *Med* median, *Min* minimum, *mRS* modified Rankin Scale, *MTL* mediotemporal lobe (amygdala, hippocampus), *N* negative, *n.d.* not done, *R* right, *titer* 1:n

**Fig. 1:**
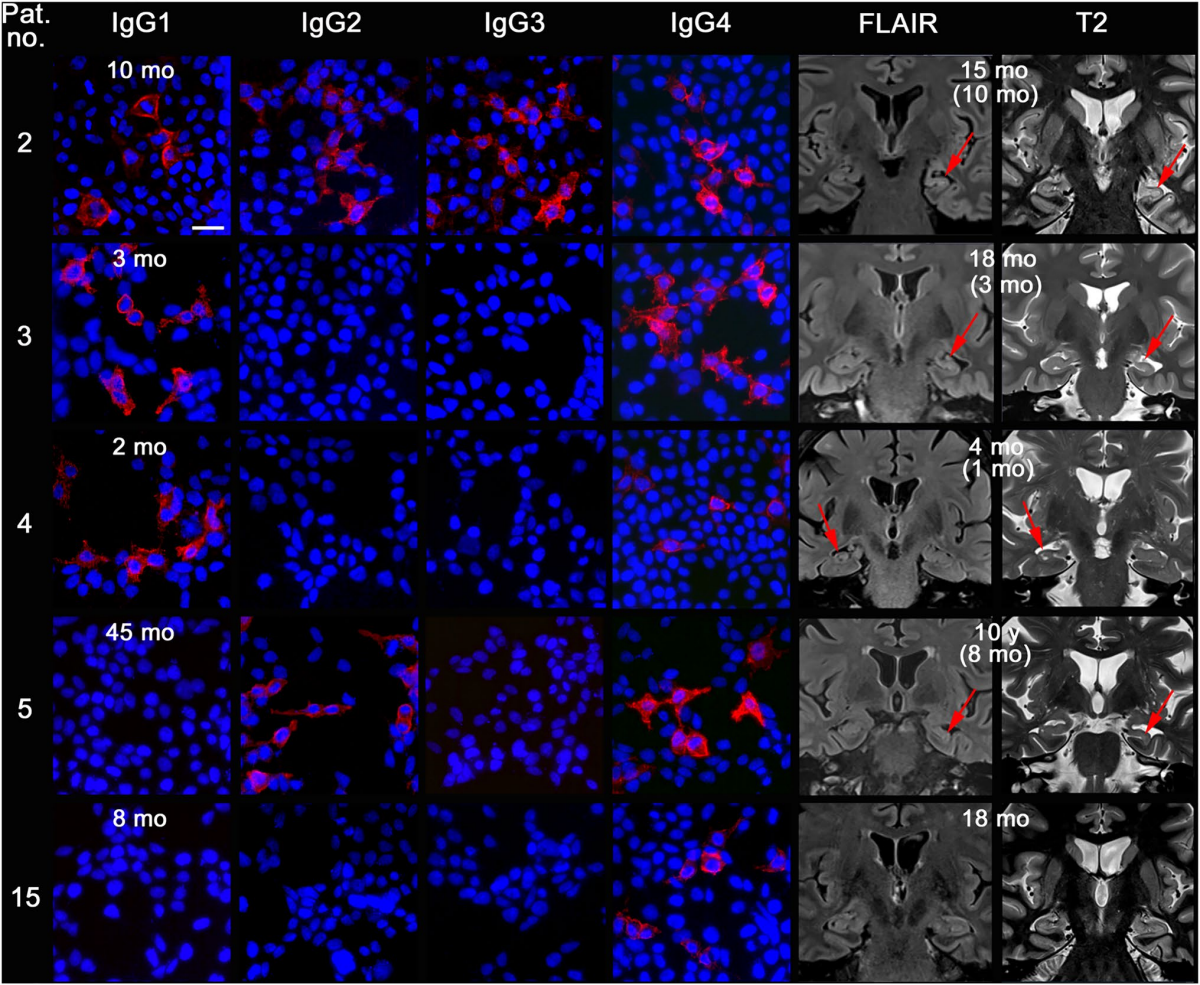
Five exemplary anti-leucine-rich, glioma inactivated 1-(LGI1-)immunoglobulin G subclass studies on HEK cells transfected with LGI1 and the patients’ most recent coronal MRI. The patient numbers in the first column are those in Table 2. MRI sections go through the hippocampi. Affected hippocampi are marked by arrows. Fluid-attenuated inversion recovery (FLAIR) images emphasize the increased signal in the affected hippocampi, while T2 images highlight the hippocampal atrophy. The time specifications give the latencies from disease onset to the respective depicted diagnostics. In the MRI columns, time specifications in brackets indicate when hippocampal sclerosis was detectable for the first time. Bar: 15 µm, valid for all cell-based assays

**Fig. 2:**
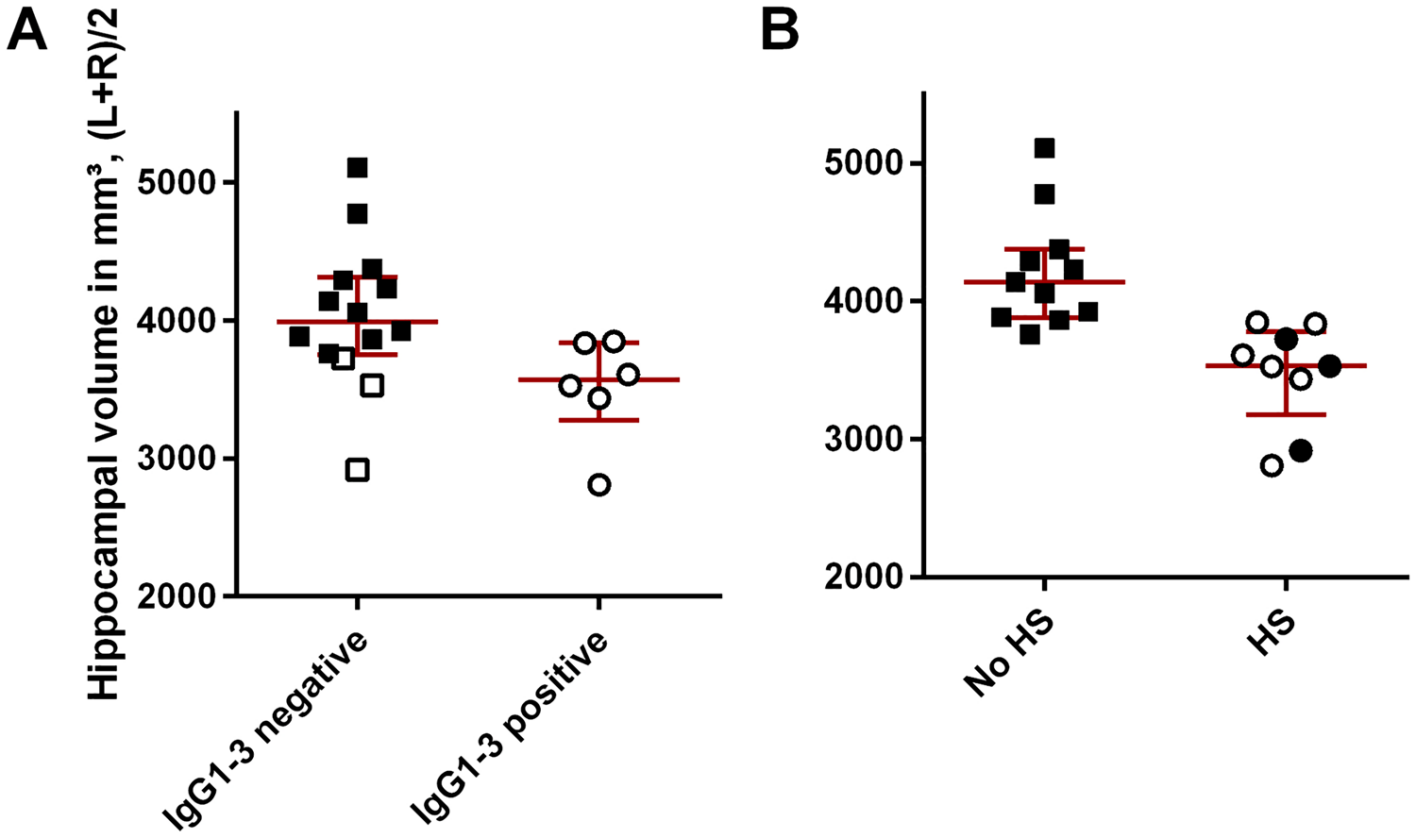
Hippocampal volumes in relation to anti-leucine rich, glioma inactivated 1-(LGI1-)immunoglobulin G (IgG) subclass status and the visual diagnosis “hippocampal sclerosis”. **A** Anti-LGI1-IgG1/2/3 status vs. hippocampal volume, *p* = 0.012. Open squares and circles: cases with the visual diagnosis of “hippocampal sclerosis”. **B** “Hippocampal sclerosis” vs. hippocampal volume, *p* < 0.001. Open squares and circles: cases with anti-LGI1-IgG1/2/3 antibodies. Statistical test: Mann–Whitney *U* Test, two-tailed. Bars: Medians and interquartile ranges. *HS* hippocampal sclerosis, *L* left side, *R* right side

Nine patients (45%) had developed HS (seven unilateral, two bilateral) at the most recent examination, which took place after a median of 31 months after disease onset (range 4–173). All nine patients had IgG4 antibodies, which were the only subclass in three; five patients had in addition anti-LGI1-IgG1 antibodies (two of them with concomitant IgG2 and 3 antibodies) and one patient IgG2 antibodies (Table 2). All six patients with anti-LGI1-IgG1/2/3 antibodies developed HS. Representative case-wise anti-LGI1-IgG subclass stainings and follow-up MRIs are shown in Fig. 1. Anti-LGI1-IgG1/2/3 antibodies were significantly more frequent in patients who later developed HS (*p *= 0.002, Fisher’s exact text, two-tailed); the sensitivity of the anti-LGI1-IgG1/2/3-subclass status predicting HS was 0.67 (95% confidence interval [CI] 0.30–0.93), the specificity 1.00 (95% CI 0.72–1.00), the positive predictive value 1.00 (95% CI 0.54–1.00), and the negative predictive value 0.79 (95% CI 0.49–0.95). Hippocampal volumes significantly differed between patients with and without anti-LGI1-IgG1/2/3 (*p *= 0.012, Fig. 2A), as well as those with and without diagnosis HS (*p *< 0.001, Mann–Whitney *U* tests, Fig. 2B). The HS had already been detected as early as 7.5 months (median; range 1.4–20.2 months) after disease onset.

The anti-LGI1-IgG1/2/3 status was significantly associated with HS (Phi=0.72, *p*<0.001) and with hippocampal volume (*r*_pbis_ = − 0.46, *p*=0.041; Table 3). Apart from that, the number of immunotherapies was significantly associated with “HS” and not with “hippocampal volume”; importantly, the association was in the inverse direction to that which had been hypothesized: more (not less) immunotherapies were associated with “HS”. Similarly, the specific LGI1 antibody index correlated with “hippocampal volume” (not with “HS”) but was again inversely correlated to the hypothesis as higher indices were correlated to larger (undamaged) hippocampi. None of the other hypothesized factors was associated with the dependent variables “HS” or “hippocampal volume”: sex, age at onset, latency from disease onset to start of immunotherapy, LGI1 antibody titer in serum or CSF (log2), latency from start of immunotherapy to follow-up MRI, or mediotemporal lesion on the earliest available MRI scan (Table 3).

**Table 3:** Bivariate associations with “hippocampal sclerosis” and “hippocampal volume”

	Hippocampal sclerosis		Hippocampal volume	
	Association^a^	*P*	Association^a^	*p*
Anti-LGI1-IgG1/2/3	**0.72**	** < 0.001**	− **0.46**	**0.041**
Sex^b^	− 0.39	0.086	0.43	0.056
Age at onset	− 0.06	0.815	− 0.24	0.309
Latency disease onset to start of immunotherapy, *n* = 19	0.17	0.487	− 0.16	0.522
LGI1 serum ab titer 1:*n*, log2	0.19	0.416	− 0.26	0.262
LGI1 CSF ab titer 1:(*n* + 1)^c^, log2, *n* = 18	0.23	0.357	− 0.05	0.844
Specific LGI1 antibody index, *n* = 18	− 0.05	0.842	0.48^d^	0.045^d^
No. of immunotherapies	0.47^e^	0.036^e^	− 0.22	0.340
Latency onset–follow-up MRI	0.28	0.238	− 0.30	0.200
MTL lesion on earliest available MRI scan	0.24	0.303	− 0.08	0.748

*ab* antibody, *IgG* immunoglobulin, *LGI1* leucine rich, glioma associated protein 1, *MTL* mediotemporal lobe (amygdala, hippocampus)

^a^Phi coefficient for associations between two binary variables, Pearson correlation or point-biserial correlation for associations with continuous variables

^b^Sex was coded as female = 1 and male = 2. The (non-significant) negative association with hippocampal sclerosis indicates that men are less likely to develop hippocampal sclerosis. The positive association with hippocampal volume indicates that men have a higher hippocampal volume than women

^c^To also include the titer “0”

^d^Please note the direction: higher antibody indices (indicating more intrathecal antibody synthesis) are associated with larger hippocampi, which contradicts the hypothesis

^e^Please note the direction: more immunotherapies are associated with a higher likelihood of hippocampal sclerosis, which contradicts the hypothesis

There was no association of the anti-LGI1-IgG1/2/3 status with verbal learning or memory (data available for 4/6 patients from the LGI1-IgG1/2/3-positive and 11/14 from the negative group after a median follow-up of 52 months, range 4 months–14 years; all *p* > 0.500, data not shown). In no patient could epilepsy be diagnosed. There was no association with the mRS (*r*_pbis_ = − 0.18, *p *= 0.436) or CASE scores (*r*_pbis_ = − 0.05, *p *= 0.830).

## Discussion

In this sample of 20 patients with anti-LGI1 encephalitis and a 100% rate of anti-LGI1-IgG4 serum antibodies, the additional presence of potentially complement activating anti-LGI1-IgG1/2/3 was the only factor that was associated with HS and reduced hippocampal volumes according to the initial hypotheses.

This fits well with the previous neuropathological observation of mediotemporal Ig deposition and C9neo expression, which indicates the functional activation of the classical complement cascade, together with neural cell death in affected humans [4, 25]. Cats with this disease exhibit the same features [22]. In the cats, a breakdown of the mediotemporal vascular tight junctions with leakage of proteins from the blood stream into the CNS explains the spatial selectivity of the disease process for limbic structures [46]. Alternatively or additionally, anti-LGI1-IgG1/2/3 could exert other downstream IgG effects such as internalization of the LGI1-ADAM22/23 complex [38]. It would need to be demonstrated how this mechanism may lead to HS.

The specificity and positive predictive value of anti-LGI1-IgG1/2/3 positivity for HS development was 100%. Sensitivity and negative predictive values were lower since three patients developed HS in the absence of anti-LGI1-IgG1/2/3. This suggests that either the test is not sensitive enough or that other factors can lead to HS.

Other analyzed factors were sex, age at onset, latency from disease onset to start of immunotherapy, LGI1 antibody titer in serum or CSF, specific LGI1 antibody index, number of immunotherapies, latency from start of immunotherapy to follow-up MRI and mediotemporal lesion on the earliest available MRI scan; none were associated with the dependent variables “HS” or “hippocampal volume”. HS was associated with more immunotherapies, which is probably not causative but rather the consequence of a more severe disease course, likely triggered by the anti-LGI1-IgG1/2/3; also, stronger intrathecal synthesis of the antibody was associated with larger hippocampal volumes, which is counterintuitive.

The MRI diagnosis “HS” is the typical expression of acquired hippocampal damage with nerve cell loss and gliosis [6]. The visual assessment acknowledges volume and signal differences in parallel to detect hippocampal damage with high sensitivity [48]. The FreeSurfer approach is objective but limited to volume measurement and was, therefore, primarily used as a control step of the visual results. Indeed, lower volumes were associated with the diagnosis “HS”, which supports the validity of the visual diagnoses (Figure 2B).

The study that is most comparable to our investigation focused on the intrathecal production of LGI1 antibodies (specifically of anti-LGI1-IgG4). Apart from some differences in methodology, the authors chose mRS and not HS on MRI as their primary outcome variable. The authors found an association for intrathecal LGI1 antibody synthesis but not anti-LGI1-IgG subclass status with mRS or with “MRI”; they did not give details on the latter [14].

Features of anti-LGI1 encephalitis suggesting a pathophysiological action of bivalent, i.e. IgG1/2/3, activity have been observed before: LGI1 antibodies were experimentally able to internalize LGI1-ADAM22/23 complexes, which is impossible for IgG4 [34, 35, 38, 45]; another study found that patients with FBDS and cognitive impairment had a higher proportion of LGI1-IgG1 antibodies compared to those with FBDS only (MRI data were not given) [45].

If our observation is confirmed in other, ideally larger cohorts, if a clinical relevance becomes evident and if more neuropathological evidence supports the complement-hypothesis, the use of complement-inhibiting compounds (like eculizumab, ravulizumab, and zilucoplan, licensed for myasthenia gravis [8]) could become a specific therapeutic option for patients with anti-LGI1-IgG subclasses 1/2/3. Simply giving more standard immunotherapies, however, does not seem to be beneficial.

### Limitations

The number of patients in this series is limited. This results from the fact that we intended to have as precise and comparably collected MRI and clinical data as possible, which are difficult to obtain in laboratory-based studies. There is the broad range of follow-up latencies but no statistical difference between anti-LGI1-IgG1/2/3-positive and -negative patients. The retrospective nature of the study may have introduced bias. To minimize it, co-authors assessing the MRIs (FGW, MM) and the clinical outcomes (AR) were blind for the subclass status of the patients. In 4/20 cases, LGI1 serum antibodies were neither confirmed by a serum or CSF neuropil pattern on the tissue-based assay nor by CSF positivity on the CBA. Limited sensitivity for LGI1 antibodies in CSF has been documented with the Euroimmun assay [28]; this, however, was not the case with serum in one dedicated study [33]. For the validity of the LGI1 serum positivity, we relied on the typical clinical presentation and anti-LGI1-IgG4 reactivity.

It is possible that our IgG subclass determination is not sufficiently sensitive; the positivity rates for the anti-LGI1-IgG subclasses lies, however, well in the range of previous studies (with the French group standing out by a particularly high rate of IgG1), see Table 1. In the present study, the negative anti-LGI1-IgG1-4 staining in sera with low titer antibodies and in CSF would speak in favor of insufficient sensitivity; other groups had the same difficulties [14, 16]. At present, there is no other method with which the CBA subclass results could be validated or improved in sensitivity.

The lack of an association of anti-LGI1-IgG1/2/3 with a poorer verbal memory outcome is probably related to the fact that the left hippocampus in the HS group was not always affected, which subsides verbal memory [49]. On the other hand, memory performance can deteriorate in this ageing population even in the absence of HS. Neuropsychological studies in autoimmune encephalitis in general, and even in the more circumscribed domain of limbic encephalitis, have been considered essential for the follow-up of patients to inform treatment decisions [50]. Systematic, group-wise studies of affected individuals, however, have been less discriminative and informative than, e.g., in the context of epilepsy surgery. For example, specific associations of autoantibody types with memory performance or other neuropsychological domains could not be found acutely or in the chronic phase [29, 30]. Reasons for this may be the small numbers of patients, the dynamic nature of autoimmune encephalitis, the right-sided, left-sided or bilateral affection within one antibody group (as in the present study) and the higher age of individuals with anti-LGI1 encephalitis with potentially concomitant and overlapping deficits [31]. Such deficits may have existed pre-morbidly, may have evolved for non-encephalitis-related reasons with age, or both. Of note, Finke et al. found correlations between some MRI measures of hippocampal subfield atrophy and domains of the RAVLT [12]. No association between the IgG subclass status and clinical outcome was reported by Muñiz-Castrillo [31]

While neuropsychological measures may, therefore, be too sensitive to detect differences in a small sample, the mRS and CASE scores may not be sensitive enough, especially in the psychological domain. Also, none of our patients developed epilepsy [36]; therefore, with this parameter, too, no difference between anti-LGI1-IgG1/2/3-positive and -negative patients could be detected.

## Conclusion

Anti-LGI1-IgG1/2/3 antibodies carry a high risk of the development of HS. The neuropathological evidence of activation of the classical complement with spatially related mediotemporal nerve cell loss in humans and in cats is a plausible link to neural death and hippocampal atrophy. HS may contribute to a poorer outcome of anti-LGI1 encephalitis, even though this could not be demonstrated in our limited number of patients. If our findings are corroborated, if the clinical relevance can be demonstrated and if further neuropathological work confirms the complement hypothesis, anti-complement-directed compounds may become useful in patients with anti-LGI1-IgG1/2/3 antibodies.

## Supplementary Information

Below is the link to the electronic supplementary material.

Supplementary file1 (DOCX 34 KB)

## Data Availability

Anonymized data not published within this article will be made available by request from any qualified investigator.
