# Supplementary material for: LGI1 encephalitis: potentially complement-activating anti-LGI1-IgG subclasses 1/2/3 are associated with the development of hippocampal sclerosis
Source: J Neurol. 2024 Aug 6;271(9):6325–35. doi: 10.1007/s00415-024-12594-9 (PMC11377613; doi:10.1007/s00415-024-12594-9)
Supplement: Supplementary file 1 — Supplementary file1 (DOCX 34 KB) [file 415_2024_12594_MOESM1_ESM.docx]

**Supplementary table 1:** Additional patient data

| **Pat. no.** | **Sex** | **Age at onset (y)** | **Syndrome** | **Onset - ab diagnostics (mo)** | **Onset – immuno-tx (mo)** | **Serum/ CSF neuropil staining** | **Immuno-tx sequences** | **Onset – earliest MRI (mo)** | **Onset - earliest MRI with HS (mo)** | **Earliest MRI lesion** | **Onset – latest follow-up (mo)** |
| --- | --- | --- | --- | --- | --- | --- | --- | --- | --- | --- | --- |
| 1 | F | 66.2 | FBDS, LE | 12.3 | 12.5 | P/N | Pr, Aza | 12.2 | 20.2 | Edema R MTL | 68.1 |
| 2 | M | 58.0 | FBDS, LE | 10.3 | 10.5 | P/n.d. | Pr | 4.1 | 10.3 | Edema L MTL | 14.7 |
| 3 | F | 22.3 | FBDS, LE | 3.3 | 1.7 | P/P | IVMP, PEX, Pr, IVIG, RTX | 1.7 | 2.8 | Edema both MTL, laminar edema L hipp | 18.1 |
| 4 | F | 75.5 | FBDS, LE | 1.8 | 1.6 | N/N | IVMP, Pr, PEX | 1.4 | 1.4 | HS R | 3.9 |
| 5 | M | 59.0 | LE | 44.7 | 43.4* | N/N | Pr, IA, IA, Aza, MMF | 6.7 | 7.5 | Edema L hipp | 172.7 |
| 6 | F | 65.0 | LE | 12.5 | 12.6 | N/N | Pr, IA, MMF | 0.1 | 12.6 | None | 137.9 |
| 7 | M | 48.3 | LE | 1.2 | 0.9 | P/P | Pr, IA, Aza | 1.2 | 6.4 | Laminar edema R hipp | 32.3 |
| 8 | F | 65.2 | LE | 14.6 | 12.0* | N/n.a. | Pr | 0.1 | 11.9 | Edema L hipp | 95.0 |
| 9 | F | 53.0 | LE | 7.7 | 7.7 | P/n.d. | Pr, IA | 0.0 | 2.9 | None | 59.0 |
| 10 | M | 72.1 | FBDS | 6.1 | 2.2* | P/P | Pr | 0.6 | n.a. | None | 5.6 |
| 11 | M | 59.1 | LE | 8.2 | 8.4 | P/P | Pr | 0.4 | n.a. | Edema R temp-lat | 30.5 |
| 12 | F | 65.6 | 2 TCS | 4.3 | No IT | P/N | None | 3.5 | n.a. | None | 8.7 |
| 13 | F | 41.5 | LE | 44.4 | 14.4* | P/n.a. | IVMP | 10.6 | n.a. | Edema R MTL | 44.4 |
| 14 | M | 61.0 | FBDS | 10.0 | 9.9 | N/n.d. | Pr | 1.5 | n.a. | None | 62.5 |
| 15 | M | 52.2 | LE | 8.5 | 10.2 | P/n.d. | Pr | -6.2** | n.a. | Edema R amygdala | 17.5 |
| 16 | M | 61.1 | LE | 15.3 | 14.8 | P/N | Pr, IA | 10.1 | n.a. | Edema R MTL | 29.8 |
| 17 | M | 57.0 | FBDS | 2.7 | 2.6 | P/P | Pr, Pr, IVMP | 0.1 | n.a. | None | 25.0 |
| 18 | M | 62.9 | FBDS | 15.1 | 0.5* | P/N | IVMP, Pr | 13.8 | n.a. | Edema R amygdala | 14.8 |
| 19 | M | 50.3 | LE | 9.8 | 10.0 | N/N | IVMP, Pr, IA | 2.0 | n.a. | Edema R amygdala | 102.9 |
| 20 | F | 57.5 | FBDS | 8.2 | 10.0 | N/N | Pr, IA | 8.0 | n.a. | None | 108.6 |
| *Med* | *9 F,*  *11 M* | *59.0* | *9 FBDS, 14 LE, 1 TCS only* | *9.1* | *10.0* |  | *2* | *1.6* | *7.5* |  | *31.4* |
| *Min* |  | *22.3* |  | *1.2* | *0.5* |  | *0* | *-6.2*** | *1.4* |  | *3.9* |
| *Max* |  | *75.5* |  | *44.7* | *43.4* |  | *5* | *13.8* | *20.2* |  | *172.7* |
| *Mean* |  | *57.6* |  | *12.0* | *9.8* |  | *2* | *3.6* | *8.4* |  | *52.6* |
| *SD* |  | *11.5* |  | *11.9* | *9.5* |  | *1* | *5.1* | *6.0* |  | *47.9* |

*In these cases, the immunotherapy had been started before transfer to our department, or no earlier serum samples were available for IgG subclass testing.

**This MRI was performed as part of the diagnostic evaluation of a depressive episode starting 2 y prior to the first tonic-clonic seizure, which led to the diagnosis of the anti-LGI1 encephalitis and is here taken as “onset”.

**Abbreviations:** Aza=azathioprine; F=female; FBDS=faciobrachial dystonic seizures; hipp=hippocampus; HS=hippocampal sclerosis; IA=immunoadsorption series; IT=immunotherapy; IVIG=intravenous immunoglobulins; IVMP=intravenous methylprednisolone pulse; L=left; LE=limbic encephalitis; M=male; Max=maximum; Med=median; Min=minimum; MMF=mycophenolate mofetil; mo=months; MTL=mediotemporal lobe; N=negative; n.a.=not applicable; P=positive; Pr=prednisolone scheme; R=right; RTX=rituximab; SD=standard deviation; TCS=tonic-clonic seizures; temp-lat=temporo-lateral tx=therapy; y=years.

**Supplementary information 1:** Immunotherapy regimens

All but one patient (no. 12) received immunotherapies: most often oral prednisolone (80 mg/d for 4 weeks, thereafter reduced by weekly steps of 10 mg/d down to 10 mg/d, which was then tapered to zero within several months at the treating physician’s discretion); intravenous methylprednisolone pulses (250-1000 mg over 3-5 days, duration at the physician’s discretion); intravenous immunoglobulins (start with 3-5 times 0.4 g/kg body weight, thereafter monthly 0.4 g/kg; duration at the physician’s discretion); plasma exchange (usually 5 treatments, each exchanging 1.0-1.5 plasma volumes); immunoadsorption (usually 10 treatments, 2.0-2.2 plasma volumes each); azathioprine (usually dosed to reach a lymphocyte count of 0.6-1.0 G/l); and mycophenolate mofetil (1-2 g/d). Patients received a median of two immunotherapies (range 0-5). For individual treatment sequences see **Supplementary table 1**.
